# Moral Injury Among Frontline Long-Term Care Staff and Management During the COVID-19 Pandemic

**DOI:** 10.3389/frhs.2022.841244

**Published:** 2022-05-11

**Authors:** Kristin A. Reynolds, Lily Pankratz, Barbie Jain, Bronwen Grocott, Lynette Bonin, Gillian King, Jordana L. Sommer, Renée El-Gabalawy, Ryan J. Giuliano, Maia Kredentser, Natalie Mota, Leslie E. Roos

**Affiliations:** ^1^Department of Psychology, Faculty of Arts, University of Manitoba, Winnipeg, MB, Canada; ^2^Department of Psychiatry, Max Rady College of Medicine, Rady Faculty of Health Sciences, University of Manitoba, Winnipeg, MB, Canada; ^3^Perley Health, Ottawa, ON, Canada; ^4^Department of Clinical Health Psychology, Max Rady College of Medicine, Rady Faculty of Health Sciences, University of Manitoba, Winnipeg, MB, Canada; ^5^Department of Anesthesiology, Perioperative and Pain Medicine, Max Rady College of Medicine, Rady Faculty of Health Sciences, University of Manitoba, Winnipeg, MB, Canada; ^6^Department of Pediatrics, Max Rady College of Medicine, Rady Faculty of Health Sciences, University of Manitoba, Winnipeg, MB, Canada

**Keywords:** moral injury, long-term care, COVID-19, clinical symptoms, qualitative, grounded theory analysis, Canada

## Abstract

**Background:**

A growing body of research highlights the experiences of moral injury among healthcare professionals during the COVID-19 pandemic. Moral injury (i.e., participating in or witnessing acts that violate one's central moral values), is associated with a host of psychological sequelae and corresponding negative psychosocial impacts. There is a lack of research examining the experiences of moral injury among those working in long-term care settings during the COVID-19 pandemic. Given the drastic impact that the COVID-19 pandemic has had on long-term care facilities in Canada, it is important to understand the experiences of moral injury among those working in long-term care settings to inform the development of effective prevention and intervention strategies.

**Objectives & Method:**

The objectives of this study were to understand the experiences and impact of moral injury among Canadian frontline long-term care workers (staff and management) during the COVID-19 pandemic. Participants (*N* = 32 long-term care staff and management working in Ottawa and Manitoba) completed in-depth, semi-structured qualitative interviews and clinical diagnostic assessments (Mini International Neuropsychiatric Interviews; MINI; Version 7.0.2) between March 2021-June 2021.

**Findings:**

The core category of our qualitative grounded theory model of moral injury in long-term care exemplified four shared types of morally injurious experiences, paired with cognitive, affective, and physiological symptom domains. Seven associated main themes emerged, contributing to the experiences and impact of moral injury in long-term care: 1) Beliefs about older adults and long-term care; 2) Interpretation of morally injurious experiences; 3) Management of morally injurious experiences; 4) Long-term care pandemic impacts; 5) Personal pandemic impacts; 6) Structural impacts in long-term care; and 7) Mental health needs and supports. Clinical assessments demonstrated anxiety disorders (*n* = 4) and feeding and eating disorders (*n* = 3) were among the most frequently classified current psychiatric disorders among long-term care workers.

**Conclusions:**

This is the first Canadian study to examine the experiences and impact of moral injury in long-term care during the COVID-19 pandemic using qualitative and clinical diagnostic methodologies. Implications and insights for screening and intervention are offered.

## Introduction

The experience of morally injurious events can be defined as “perpetrating, failing to prevent, bearing witness to, or learning about acts that transgress deeply held moral beliefs and expectations” (1); p. 697. Feelings of betrayal by authorities with shared core moral values is also a prominent feature of moral injury (2). The term moral injury, though initially developed with a focus on military personnel (3), is increasingly being applied to healthcare professionals, in recognition that the experiences and psychological sequelae that were previously labeled as burnout were insufficiently explained by this term (4). Further, interventions designed to prevent and treat burnout in healthcare professionals were overwhelmingly being rendered as ineffective, leading researchers and clinicians to consider the applicability of moral injury in this population (5, 6).

Seminal research by Litz et al. (1) provided a foundation for understanding moral injury and associated psychological sequelae, including posttraumatic stress disorder (PTSD), self-harming and self-handicapping behaviors, and hopelessness. Other studies have since highlighted additional impacts of morally injurious experiences, such as negative impacts on social bonds (7), psychospiritual development, psychosocial functioning (8), and increased risk for depression, anxiety, and suicidal ideation (9). Researchers posit that the objective severity of a morally injurious event is less important than the subjective appraisal of the experience (10). A recent study highlighted the role of rumination as a risk factor for poor mental health problems among military personnel who experience moral injury (11). Self-compassion has been identified as a protective factor buffering against the adverse consequences of moral injury (12). As research topic in great need of further exploration, there is debate in the extant literature concerning the amount of overlap between expressions of moral injury and PTSD, in addition to the degree to which gold standard treatment approaches for PTSD adequately address moral injury (13). In distinguishing PTSD from moral injury, an important study by Bryan et al. (14) notes two distinct but associated symptom profiles. The PTSD symptom profile includes key symptoms such as exaggerated startle response, trauma-related memory impairment, flashbacks, nightmares, and insomnia. In contrast, the moral injury profile includes the key symptoms of guilt, shame, anger, anhedonia, and social withdrawal (14). However, it is important to note the continued view that these constructs are still overlapping even though distinct features are noticed. Changes in DSM-5 PTSD nomenclature have been said to accommodate some conceptual components of moral injury; however, may not capture the full experience (15). One clear finding across studies indicates that those who have been exposed to potentially morally injurious events are at a greater risk of developing negative psychological symptoms than those who are not exposed (9, 13). Regarding treatment approaches for moral injury, several studies have found support for the use of evidence-based psychotherapies designed to treat PTSD including prolonged exposure therapy (PE) and cognitive processing therapy (CPT) as leading to reductions in trauma-related guilt (13). Further, there is promise indicated for the application of Ehlers and Clark's (16) cognitive model of PTSD in addressing features of moral injury-related PTSD (17).

Particularly during the COVID-19 pandemic, when the divide between ideal practice and actual practice in healthcare has widened farther than perhaps ever before, it is important that we consider moral injury in healthcare workers (18). One recent study investigating moral injury among health care professionals during the COVID-19 pandemic noted an increase in moral injury throughout the duration of the pandemic (19). Psychological (e.g., depression), physiological (e.g., pain), cognitive (e.g., difficulty concentrating, reduced processing speed), behavioral (e.g., insomnia, isolation, self-medication with alcohol and substances), and emotional responses (e.g., guilt and shame) have been noted across studies on moral injury prior to and during the pandemic (19). Social support (smaller networks and lower perceived support), marital status (widowed, divorced, never married), age (younger), health care profession type (nurse), and proximity to COVID-19 patients (direct experience caring for patients with COVID-19) have all been associated with increased moral injury symptoms and a greater likelihood of functional impairment (19). In several recently published first-person accounts, healthcare professionals working in the United States have compared their experiences during the COVID-19 pandemic to military contexts. For example, Ramnath (20); p. 1325 notes:

“My thoughts turned to images of war-weary soldiers adapting through depersonalization and numbing. I could not shrug off the gnawing dread while driving to the hospital, devolving into utter helplessness as I walked through the ICU, knowing that most of my patients would die no matter how intense my efforts.”

Similarly, in an editorial on the epidemic of moral injury in healthcare workers during COVID-19, Dean, Jacobs, and Manfredi (21) described:

“As with soldiers in war, we know that as soon as we stop doing, we will start feeling. The deferred processing of grief and trauma and betrayal-for the patients we've lost, the tragedy we've witnessed, and the risks foisted on us by failures and organizational foresight-will threaten to overtake us” (p. 385).

Authors of this work go on to acknowledge the challenges that many healthcare professionals face in acknowledging and accessing mental health treatment, noting, “Health care workers have learned that vulnerability—saying, “I need help”—is yoked to shame, not courage. For physicians, especially, too many would rather die than submit to the trauma of admitting helplessness or weakness” (21); p. 385.

Long-term care staff and management, largely employed in public and private settings that fall outside of formal healthcare systems, have been a relatively understudied group in terms of morally injurious experiences throughout the COVID-19 pandemic. This is especially important as long-term care facilities have experienced one of the biggest impacts associated with the COVID-19 pandemic, with over 81% of Canada's COVID-19 deaths occurring in long-term care (22). Rates of COVID-19-related deaths in Canadian long-term care settings are significantly higher when compared to other long-term care facilities across the globe (23). Canada has a two-tiered system of funding for long-term care facilities, with some being funded publicly (46%) and others privately (56%), with little regulation and oversight, and great variation in quality and access to care (24). Before COVID-19, Canadian researchers highlighted the disparity of care between non-profit and for-profit care homes, with for-profit facilities in Ontario and British Columbia receiving significantly higher rates of complaints compared to non-profit facilities (25). Another study found that for-profit homes provide significantly fewer hours of care compared to non-profit homes (26). In the 2019 Report *Caring in Crisis*, created by the Ontario Health Coalition, extreme staffing shortages, and how this shortage has created more dangerous, rushed, and stressful working conditions for all workers is highlighted (27). The COVID-19 pandemic has drawn attention to the under-funded and resourced, under-prepared, and poor living and working conditions in vital care facilities housing a large proportion of Canada's aging population (28). Mixed-methods research by our group has surveyed over 60 long-term care staff in Manitoba and highlights significantly elevated levels of perceived stress and caregiver burden among this population (29). Further to this, a recent quantitative study conducted by Brady and colleagues (30) in Ireland found *nursing home* staff reported high prevalence estimates of both posttraumatic stress and moral injury during the pandemic. Authors also noted that clinical staff experienced higher rates of moral injury compared to non-clinical workers. Researchers in the United States found that roughly half of health care workers in their sample endorsed experiencing a morally injurious event over a 90-day period during the pandemic (9). A qualitative research study conducted by French et al. (31) examined the experience of moral injury among health staff in the UK, highlighting the experience of abandonment as betrayal. The experience and impact of moral injury appears to be wide-reaching, with recent research supporting vicarious effects of trauma and moral injury on mental health professionals supporting frontline health and social care workers during the COVID-19 pandemic (32).

There are no studies that have examined the experiences and impact of moral injury in frontline healthcare professionals working in long-term care settings in Canada during COVID-19. In the face of this pandemic, frontline healthcare providers, and long-term care staff and management, are at great risk for moral injury. Long-term care providers may be at increased risk due to working long shifts with little opportunity for breaks or sleep before starting another shift; lack of time to process events that occurred during their work-shift; reduced availability to provide desired care due to decreased resources; lack of clear guidance or training on COVID-19 protocols; and lack of available protection from the virus, putting their health in danger and leading to increased risk for disease exposure (33, 34). A recently published editorial on moral injury in frontline workers during COVID-19 noted five risk factors for moral injury: 1) Loss of life to a vulnerable person (e.g., older adult); 2) Leaders perceived not to take responsibility for the morally injurious event or are not supportive of staff; 3) Staff feel unaware or are unprepared for emotional/psychological consequences; 4) If potentially morally injurious events occur at the same time as other traumatic events; 5) Lack of support following the event (35). Further impacting the mental health of staff in long-term care facilities are physical distancing requirements, which may limit the emotional and physical support or coping strategies that staff may have practiced prior to the pandemic, as well as the impact of observing deaths that are often uncontrollable, and due to physical distancing requirements, witnessing the pain of family members who were not able to spend time with their loved ones prior to their deaths.

Currently, there are no manualized approaches to diagnose, prevent, or treat moral injury-related mental health difficulties (35). The overarching aim of this research was to understand the experiences and impact of moral injury in long-term care staff during the COVID-19 pandemic. By understanding the experiences and impact of moral injury, we can start to develop interventions to prevent and treat moral injury in this population, a field in need of much development at this vital time.

## Method

### Recruitment

We recruited long-term care workers in several ways, with the aim of understanding the experiences of a broad sample of long-term care employees, including those who recently began employment in this setting, as well as those who have been employed in this setting for a longer time-period, those working within various positions, and those working in settings with a range of funding models. Recruitment, screening, and data collection for qualitative interviews and clinical assessments took place between March 2021 – June 2021. We contacted respondents from prior survey research by our group examining the experiences of long-term care staff working during the COVID-19 pandemic. Additionally, we recruited participants from a technical college in central Canada with a newly developed Health Care Support Worker Program, developed provincially as a rapid response to employment needs during the COVID-19 pandemic. Participants were also recruited from long-term care facilities across Manitoba through general advertisements as well as targeted advertisements through a seniors and veterans' long-term care facility in Ottawa. Interested participants responded to an online study advertisement titled: Moral Injury in Long-Term Care.

### Procedure

Once participants indicated interest in participating in the study, the research coordinator scheduled eligibility screening. During this initial meeting, the research coordinator verified whether the participant was 18 years of age or older, and if they were currently employed in a long-term care facility. Additional inclusion criteria included having access to a computer/smart phone, Internet access, and English-language fluency. Litz et al. (1) definition of moral injury was reviewed with participants during initial screening, consent, and at the outset of the qualitative interview, with all participants endorsing one or more morally injurious experiences. Upon meeting eligibility requirements and endorsing agreement to participate, participants completed an online informed consent form and background questionnaire package via Qualtrics. Participants received a $25 gift card honourarium for completing the qualitative interview and an additional $25 gift card honourarium for completing the clinical assessment (MINI).

#### Background Questionnaire

The background questionnaire contained information on age, gender, level of education, occupational status, marital status, ethnic and cultural origin, current self-rated physical and mental health, current mental health diagnoses and help-seeking, and prior mental health diagnoses and help-seeking. Information on long-term care employment was also collected, including position type and training. This information was collected to help us to characterize our sample.

#### Semi-structured Qualitative Interview

Following completion of the background questionnaire package, a meeting time was scheduled with participants to complete the semi-structured qualitative interview and Mini International Neuropsychiatric Interview (36). MINI Version 7.0.2. At the outset of the qualitative interview, moral injury was defined as “*perpetrating, failing to prevent, bearing witness to, or learning about acts that transgress deeply held moral beliefs and expectations”* *(**1**)*. We asked participants about their work in long-term care during the COVID-19 pandemic; their connection to and meaning derived from working in long-term care; training received to pursue work in long-term care; main challenges faced in long-term care during COVID-19; experiences of moral injury in long-term care; ways of coping with moral injury and associated symptoms; and needs for mental health supports. Interviews were completed by the first author (Dr. Kristin Reynolds) and her trained team of research assistants. Interview time completion ranged from 45–75 min. Interviews were completed via video-conferencing software (Zoom Professional), audio-recorded, and transcribed for further analysis.

#### MINI

The Standard Adult MINI English Version 7.0.2 for DSM-5 (36) was administered by the first author and her trained team of research assistants, to assess 17 of the most common mental health disorders. All research assistants completed formal training and certification from the Harm Research Institute prior to commencing interviews. MINI diagnoses were made by the trained and certified research assistants, with consultation by the first author, who reviewed and finalized all MINI diagnoses. During MINI facilitation, participants respond with “Yes” or “No” to questions asked by the administrator and the administrator circles the corresponding response on the interview. When needed, the administrator may ask for clarification or examples from the participant. The MINI takes approximately 15 min to 1 hour to administer, depending on the number of mental health symptoms an individual is experiencing.

### Analytic Approach

Quantitative findings from the background questionnaire were analyzed using descriptive statistics. We employed constructivist grounded theory methodology (37) in our analysis of qualitative interviews. Constructivist grounded theory can be used to generate theory or develop a rich description and a deeper understanding of phenomena. Coding employed the constant comparative method, and three interconnected phases of initial, focused, and theoretical coding (37). Initial coding consists of labeling each line of the interview transcript (line-by-line coding) with the purpose of staying grounded in the data and understanding key actions and processes. Focused coding involves collapsing initial codes into larger, more comprehensive themes and sub-themes. Theoretical coding allows for connections to be drawn between themes and sub-themes in the development of a grounded theory model. Field notes and analytic memos were documented immediately following qualitative interviews, with the objective of field notes being to capture observations and descriptive data of participant interactions, and the aim of analytic memos being to expedite the data analytic process. Reflexivity practice (acknowledging lenses and life experiences of interviewers/analysts that influence the co-construction of the resulting grounded theory model), negotiated validity (individual initial and focused coding by lead author and three research assistants, developing theoretical model based on consensus), and audit trail (clear documentation of data analytic procedures) were applied to achieve qualitative rigor (37, 38). NVivo was used to assist with data organization (39). Recruitment continued until we reached theoretical sufficiency in our analyses, in which additional interview data did not reveal novel thematic dimensions (40).

## Findings

### Sample Description

We completed in-depth qualitative interviews and MINIs with *N* = 32 long-term care staff, *n* = 21 from Manitoba, and *n* = 11 from Ottawa. We heard the voices of *n* = 26 female and *n* = 6 male long-term care staff with a range in position type within long-term care settings (i.e., management, recreation, nurse, health care aide, physician, health care support worker, spiritual care, dietician, and social worker). The highest representation in our sample was among participants who self-reported position types of management, director, and administration (28%), recreation staff (19%), nursing staff (19%), and health care aids (16%). Though long-term care is often described as having female-dominated workforce, male staff and management are under-represented in our research. Further, our sample was highly educated, with all participants reporting involvement in post-secondary education (university or college). Most participants (72%) reported European cultural origins, with participants from racialized backgrounds being under-represented. A large proportion of our sample (72%) reported having a current mental health problem and current mental health service use (75%). Please see (Tables 1–3) for a detailed description of participant characteristics including demographics, workplace characteristics, self-reported health status, and health-related service-use.

**Table 1:** Demographic characteristics of (*N* = 32) frontline long-term care employees.

**Province**	N (%)
Manitoba	21 (65.6)
Ontario	11 (34.4)
**Age, M (SD)**	39.2 (13.0)
**Gender Identification**	
Female	26 (81.3)
Male	6 (18.8)
**Highest level of education**	
University undergraduate degree	14 (43.8)
University master's degree	8 (25.0)
College/trade school	5 (15.6)
Some university	5 (15.6)
**Marital status**	
Single	13 (40.6)
Married	10 (31.3)
Common law	7 (21.9)
Divorced	2 (6.3)
**Ethnic and cultural origins**	
European origins	23 (71.9)
African origins	3 (9.4)
Asian origins	4 (12.5)
Caribbean origins	1 (3.1)
Indigenous origins	1 (3.1)

**Table 2:** Workplace characteristics of (*N* = 32) frontline long-term care employees.

**Long-term care position type**	N (%)
Management, director, administration	9 (28.1)
Recreation	6 (18.75)
Nurse	6 (18.75)
Health care aide	5 (15.6)
Physician	1 (3.1)
Health care support worker	1 (3.1)
Spiritual care	2 (6.3)
Dietician	1 (3.1)
Social worker	1 (3.1)
**Occupational status**	
Full time	19 (59.4)
Part time	12 (34.4)
Casual	2 (6.3)
**Work in more than one long-term care center**	
Yes	4 (12.5)
No	28 (87.5)

**Table 3:** Health characteristics of (*N* = 32) frontline long-term care employees.

**Self-reported physical health**	N (%)
Good	13 (40.6)
Very good	10 (31.3)
Excellent	6 (18.8)
Fair	3 (9.4)
**Self-reported mental health**	
Good	15 (46.9)
Very good	10 (31.3)
Fair	4 (12.5)
Excellent	3 (9.4)
**Previous experience of mental health problem**	
Yes	18 (56.3)
No	14 (43.8)
**Type of previous mental health problems**	
Anxiety and depression	8 (37)
Anxiety	4 (12.5)
Depression	4 (12.5)
Bulimia nervosa	1 (3.1)
Psychosis	1 (3.1)
**Previous mental health service use**	
Yes	17 (53.1)
No	15 (46.9)
**Current mental health problem**	
Yes	23 (71.9)
No	9 (28.1)
**Current mental health service use**	
Yes	24 (75.0)
No	8 (25.0)

### Grounded Theory Framework

**Figure 1:**
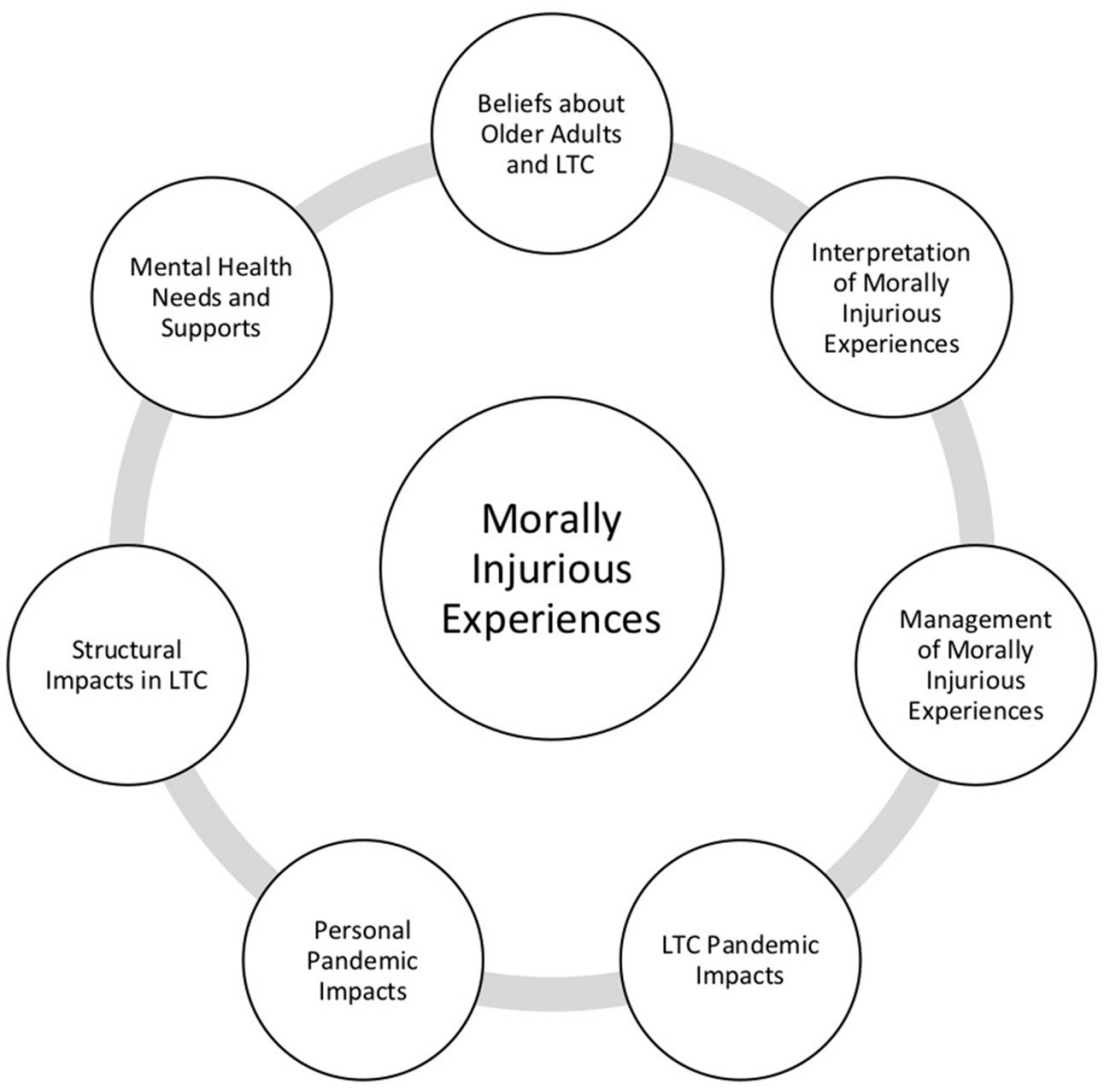
Grounded theory model of moral injury in long-term care (LTC).

Please see Figure 1 for a visual representation of our grounded theory model of moral injury in long-term care. Please see Table 4 for additional quotes supporting themes within the grounded theory model. At the center of our model, the core category emergent from qualitative interviews was morally injurious experiences. Participants described a felt sense of knowing what was best for the resident and being unable to act on this knowledge. All participants in our sample described morally injurious experiences, with specific situations falling within one of the following four sub-themes: 1) Watching residents' cognitive functioning decline due to loss of social interaction and activities; 2) Feeling disconnected from residents outside of one's immediate unit; 3) Enforcing family visitation restrictions when residents were ill and/or dying; and 4) Working with staff who were not obtaining consent from residents prior to providing care (e.g., feeding, moving, toileting). In describing many of these experiences, one participant noted:

**Table 4:** Mini international psychiatric interview diagnoses of (*N* = 32) frontline long-term care employees.

**Meeting DSM-5 criteria for past disorders**	(*N =* 17)
Major depressive episode	15
Panic disorder	2
**Meeting DSM-5 criteria for current disorders**	(*N =* 9)
Major depressive episode	1
Alcohol use disorder	1
Generalized anxiety disorder	1
Social anxiety disorder	2
Agoraphobia	1
Binge eating disorder	2
Bulimia nervosa	1

“*I mean, I would say, well, the whole experience that I've had during the pandemic kind of felt like that* [moral injury]. *Wanting to do more, wanting to get residents to visit with their loved ones, wanting to get them out to do things, and then just not being able to, obviously, because of safety.”* (P. 6).

These experiences were related to a host of psychological symptoms, which we thematically categorized into three sub-themes: 1) Cognitive (i.e., loss of control, overwhelm, perception of self as “failure,” rumination, loss of respect or faith in colleagues and authorities); 2) Affective (i.e., guilt, shame, irritation, anger, sadness, hopelessness, anxiety, loneliness, grief, numbness); and 3) Physiological (i.e., nausea, increased heart rate, agitation/tightness, tingling sensations, impaired sleep).

Related to this core category were seven emergent related main themes, including: Beliefs about older adults and about long-term care; Interpretation of morally injurious experiences; Management of morally injurious experiences; Long-term care pandemic impacts; Personal pandemic impacts; Structural impacts in long-term care; and Mental health needs and supports. These main themes progress from what appeared to be most central to the moral injury experience, what we conceptualized as driving the experience and impacts of moral injury (Beliefs about older adults and long-term care; Interpretations of morally injurious experiences; Management of morally injurious experiences) to pandemic, personal, and structural impacts of these experiences; and finally, to the support that is needed to help long-term care staff in their management of these challenging experiences.

#### Beliefs About Older Adults and About Long-Term Care

Apparent from our analyses was that most participants held longstanding strong, positive, beliefs about older adults, which led to a sense of meaning, purpose, and engagement in their work. These beliefs appeared to envelop challenging work-related experiences during the pandemic, including morally injurious experiences. At times, these deeply held beliefs and values allowed long-term care staff to develop a voice of empowerment, protection, and support during challenging situations in long-term care, thus potentially alleviating their experiential impact of morally injurious events. These beliefs were also described by participants as promoting long-term care staff to do more, to work harder and longer, often at their own expense. Participants described long-term care work as a “calling” or as “my duty,” referring to residents as “my residents,” “my people,” “my family.” These experiences of working harder at one's own expense and seeing one's employment as one's only and true “calling” may heighten the impact of morally injurious events (33–35). In describing their connection to their work in long-term care, one participant noted:

“*Even though there's some days where I'm just like, “Why am I doing this?”, I think more than ever this experience has really solidified for me my own sense of vocational calling. That this is where I need to be. This is what I need to be doing because I do highly identify with my work, and I do derive great meaning and purpose from the work that I do*” (P. 13).

#### Interpretation of Morally Injurious Experiences

Staff interpreted morally injurious events in three main ways, which had a likely impact on their experience of moral injury and associated symptoms: 1) Responsibility placed on self “I failed this family”; 2) Responsibility placed on others “I watched it happen, they did not meet the resident's need”; or 3) Responsibility was externalized “This is COVID.” Apparent from our analyses was that externalizing responses were not commonly discussed, and that staff working in administrative or managerial positions more frequently endorsed externalization of responsibility as an interpretation of the morally injurious event, compared to workers in direct, daily contact with residents, who placed responsibility on the self and less frequently, on others.

#### Management of Morally Injurious Experiences

Participants described four important ways in which they responded to morally injurious events: 1) “Finding and using my voice”; 2) Identifying creative solutions “You get creative”, “You find gray areas”; 3) Working harder “I meet resident and family needs in any way that I can”; and 4) Distancing/Rationalization – an attempt to avoid the event and associated emotional and cognitive impacts. Participants voiced coping with events through avoidance (e.g., alcohol and drug use, eating), acceptance, connecting, exercise, engaging in creative outlets, and engaging in spiritual or faith-based practices. Many participants voiced the importance of connecting particularly with colleagues who have shared workplace experiences. For example, one participant noted:

“*I have a really good team and I think there's huge value in a really great team. What I mean by this is that they show up, they work hard, they work extra hours. They don't complain, but they play and they're funny and they're fun and they cry with you and they're angry with you and they're laughing with you”* (P. 26).

#### Long-Term Care Pandemic Impacts

There were several pandemic-related changes which further impacted upon long-term care staff experiences with moral injury: 1) Changes in workload, roles, and responsibilities; 2) “Change saturation” – feeling overwhelmed by dynamic and evolving changes to long-term care policies and procedures; 3) Lack of visitation; and 4) Use of Personal Protective Equipment (PPE; pain and discomfort, loss of ability to communicate and connect with residents). In describing many of these challenges, one participant noted:

“*I have to think now, there's been so many changes, so maybe one day you're* [doing] *what you're told, all these masks or this type of equipment is good enough. You don't need anything special. A few days later, oh, no, that's not standard practice anymore. It's not good enough. You have to have N95 masks. You have to have these type of goggles or eyewear and you know, you have to gown and glove, you know, and then the next day. Oh no, you don't need to do that. So it's just to try and keep up with that sort of thing”* (P. 10).

#### Personal Pandemic Impacts

Participants shared their experience of continued sacrifice throughout the pandemic, and the irritability, frustration, anger, and sadness that they felt seeing others in the country return to normalcy when they felt “trapped” due to the added safety measures that they needed to follow to protect the most vulnerable populations in long-term care. Participants described personal impacts in four key areas: 1) Less time (working more); 2) Reduced contact with friends and family (limited to ensure resident safety); 3) Restriction of individual activities (to ensure resident safety); and 4) Losses (personal and workplace losses and grief). The following participant quotes depict the sacrifices made by long-term care workers in order to maintain an elevated level of safety for their residents.

“*We've always maintained a zone more intense than what we needed to. So when restrictions came out on gathering sizes and all of that, we were already at that small size beforehand. So certainly living in the red zone pretty much the entire time has been a sacrifice.”* (P. 29).

#### Structural Impacts in Long-Term Care

Participants described the structural forces impacting long-term care work, which further impacted upon their experiences of moral injury. These impacts included: 1) Resources and staffing (short-staffed, lack of resources); 2) Hierarchy and positioning; 3) Training and education (variable); and 4) Restrictions imposed upon long-term care facilities by the Minister of Health/Minister of Long-Term Care.

In describing resource and staffing impacts, one participant noted:

“*I always knew it was a hard job, I can understand why, but we just need we need more workers. We need more hours to really give the residents what they need. From toileting to feeding, dressing, bathing, showering, it's a lot. Your day is full. It's over full”* (P. 9).

In describing many of the structural impacts faced within long-term care settings, another participant described:

“*This is a totally broken system. And it has only managed to get this far because of the heroics of the people who work in it every day. And what COVID has done is turn the lights on. It's no worse or no different or no better than it was before COVID. It's the same. And it has always just worked because of the incredible commitment of mostly a female employee workforce who keep giving and giving and giving of themselves because they care. It's just a totally broken system. COVID has accentuated the challenges of inadequate funding or funding put in the wrong places. And money is not always the solution, but using it properly is. Training, education, which again relates to the female workforce piece*.” (P. 14).

#### Mental Health Needs and Supports

Overall, participants reported a host of unmet mental health needs within long-term care settings during the COVID-19 pandemic. Participants noted the importance of peer support/peer integration – having the support of those who are “going through it” and “truly understand what these experiences are like.” For example, one participant described, “*For me it's expressing my challenges, it's someone to talk to that gets you*” (P. 28). Online interventions (self-directed and virtual group options) with clinician involvement (Clinical Psychologist) were also described as important in order to increase accessibility. Participants also described financial barriers to accessing mental health services. For example, one participant noted, “*Money is tough. I have maybe five sessions covered, but it's always kind of like in the back of my head, well, how much does it cost when it's not covered?”* (P. 8).

One participant described issues with online mental health supports at this time for workers, and the structural constraints associated with taking time for mental health care:

“*One thing that came out of all of the online webinars is that we were just bombarded with, you know, mental health webinars and do this do that for your mental health. And suddenly everybody had something online for your mental health. But the thing is, we don't have time for this. And, you know, any time you take for yourself, there's going to be payback. If you take your day off, you're just going to have a day more of work when you come back”* (P. 25).

### Mini International Neuropsychiatric Interview (MINI)

With the aim of adding clinical nuance to our understanding of the experiences and impact of moral injury in long-term care, we completed MINI evaluations with our qualitative sample of long-term care staff and management. Table 5 indicates participant diagnoses of DSM-5 past and current disorders according to MINI administrations with *N* = 32 participants. The most prevalent current psychiatric diagnoses included anxiety disorders (generalized anxiety disorder, *n* = 1; social anxiety disorder, *n* = 2; agoraphobia, *n* = 1); feeding and eating disorders (binge eating disorder, *n* = 2; bulimia nervosa, *n* = 1); major depressive episode, *n* = 1; and alcohol use disorder (*n* = 1). Past psychiatric diagnoses include major depressive episode (*n* = 15) and panic disorder (*n* = 5). Apparent from our MINI assessments was that most participants did not endorse the first screening question of the PTSD module of the MINI inquiring about experience of *an extremely traumatic event*, which meant that they did not continue with this module of assessment. Findings in this area may add further support for moral injury as a unique and separate phenomenon.

**Table 5:** Additional qualitative responses – moral injury in long-term care.

**Theme**	**Direct quote (Participant ID)**
**Core category: morally injurious experiences**	“*The one that comes really to my mind is having to say no. Having to say no to something that should be very natural. Having to say please do not hug that person. Please do not shake their hands. Please do not talk to them. Having to say no to people wanting to see their loved ones and no to residents wanting to see their families. I just don't feel it's fair and natural in my role as a social worker”* (P. 25).
**1) Beliefs about older adults and long-term care**	“*I'm a person of faith. I believe that I was put into long-term care for a reason, and I was brought into healthcare for a reason and maybe some days I don't understand why. What started off as a fluke has turned into a purpose*” (P. 11). “*I care deeply about what I do. And being in this organization has allowed me to build a team and create change that aligns with my moral compass. I'm doing something that's bigger than myself and influencing change in places that are bigger than myself”* (P. 14). “*I was drawn into nursing because I feel that it's a career that gives one's life a purpose, you know, going to work every day, providing the best possible care to our residents and our patients and just being there for individuals in their most vulnerable moments in their life gives a special meaning to one's own life. So it is that innate motivation to help others and be there for others that really pushed me to become a nurse”* (P. 16).
**2) Interpretation of morally injurious experiences**	“*I feel like I failed her [resident's wife]. I failed that family [resident's family] because I wasn't able to continue that lifestyle for him [resident]. And their wishes. So that's hard”* (P. 3). “*I couldn't imagine anything that I could actually do to alleviate the pain that she was in. It was quite difficult to just sit there and listen to her in pain. Thinking that there might be something I could do. It was my direct orders just to try and take her mind off of it rather than directly intervene in any way. And so. It was mainly just a discomfort at seeing her in pain and not being able to do anything about it”* (P. 4).
**3) Management of morally injurious experiences**	“*Luckily, our team is very supportive of each other and we're you know, we can rely on each other. We've had a lot of venting sessions. We've had times where we're like, “OK, should I just throw my keys on the table and walk out because this is ridiculous?” But I think it ultimately comes down to faith and the support of our team. Our team, we know we have each other's back”* (P. 11). “*I would sometimes visit other residents and, you know, make sure that they're OK as well, especially during the pandemic, because I find we don't have as much as we need. So, like, whether can I get you something? Like a resident can't get out of the unit to get something, whether I could get that for them or, you know, I would send their laundry up, you know, for them to get labeled as an example, you know, things like they're physically not able to do, but they might not have a family to help them do it. And, you know, it's not my job description, but I want to still do it. And then if I have the extra time, then I'm trying to find ways to help residents in any way I can”* (P. 2).
**4) Long-term care pandemic impacts**	“*Sometimes you have lack of time with the residents, dealing with them, because you are behind more. That's what, that's what the reality at the time of the pandemic. And then when you feel like, like that, you feel bad. So I did overtime without pay. That's what happened to me, especially if more was needed”* (P. 12). “*Okay, well, one of the challenges, I would say, is having to basically worry about every single thing that you do when you're working there, so it's not violate any of the health restrictions to put yourself or other people at risk. There are many rules and regulations that are changing every day as to the correct conduct and keeping residents and yourself safe. And so that's something that is difficult to manage and difficult to keep track of”* (P. 4).
**5) Personal pandemic impacts**	“*In the beginning, I saw my partner and my mother and that was about it. And then one of my best friends had a baby in November. So then a personal sacrifice of not getting to see her and spending my visits with her, sitting outside of our house and talking to her through the front door with her brand new baby”* (P. 23).
**6) Structural impacts in long-term care**	“*And it made me feel really unsafe and really questioning why I wanted to work for some place that didn't value the safety of the employees. Not my job in general, but why would I want to work for the* [name of health authority] *if the* [name of health authority] *doesn't value my safety?”* (P. 24).
**7) Mental health needs and supports**	“*While I'm thankful EAP programs exist, I've never used them because I know managed care cuts out after like five or six sessions. And when you're dealing with a marathon long situation, there's only like a little bit of benefit. Right? Like you can get some coping skills or strategies, but you know that managed care cuts out after session five or six”* (P. 13).

## Discussion

This is the first Canadian study to explore the experiences and impact of moral injury in long-term care staff and management during the COVID-19 pandemic. Qualitative interviews with 32 long-term care workers in two Canadian provinces highlighted a range of morally injurious experiences and associated cognitive, affective, and physiological symptoms. Main themes related to experiences and impact of moral injury among long-term care staff and management included: 1) Beliefs about older adults and long-term care; 2) Interpretation of morally injurious experiences; 3) Management of morally injurious experiences; 4) Long-term care pandemic impacts; 5) Personal pandemic impacts; 6) Structural impacts in long-term care; and 7) Mental health needs and supports. Each participant in our study readily provided at least one experience of moral injury in their workplace since the start of the pandemic. Although the focus of this research was not to demonstrate alignment with or generalizability to the five factors of moral injury in frontline workers (35), interestingly, participants described the impact of each of the five factors throughout their qualitative interviews. Helplessness is a key component of each of the factors identified in Williamson's editorial; a component which was discussed time and time again by long-term care providers: watching a resident pass away alone, not feeling supported by management, feeling unprepared, experiencing personal trauma or loss, and lack of support after a potentially morally injurious event. This finding concerning the importance of helplessness maps on to prior research examining moral injury in military personnel who found that problem-focused rumination is associated with psychological distress following a morally injurious experience (11). Future research is needed to examine the role of helplessness or problem-focused thinking on psychological symptom severity in long-term care workers and health professionals who have experienced morally injurious events. Although moral injury has been extended to populations outside of military personnel, there are important contextual and experiential differences between these populations, which necessitate further examination.

The most frequently identified current DSM-5 psychiatric disorders as assessed by the MINI included anxiety disorders, feeding and eating disorders, and mood disorders. Interestingly, no participant met full criteria for acute stress disorder or PTSD. Though this is somewhat surprising given the historical link between PTSD and moral injury; findings from recent research highlight two distinct yet associated symptom profiles and argue the importance of recognizing moral injury as a unique and separate phenomenon (14). Apparent from our analyses were that most participants did not endorse the first screening question of the PTSD module of the MINI inquiring about experience of *an extremely traumatic event*. It is possible that the language of *extremely traumatic* event did not fit with the types of experiences endorsed by participants, even if some of the same posttraumatic stress symptoms may have applied. Further to this, if participants did respond affirmatively to the first screening question of the PTSD module, they denied the subsequently presented avoidance-related symptoms, ending the module for them at that point. It is possible that participants were not able to avoid much of their stressful experiences within this context, as they continued to work within long-term care during the pandemic. Thus, the timing of data collection (March-June 2021) may have impacted upon participants' abilities to reflect on whether their morally injurious experiences were indeed traumatic to them, as they may have been in a mode of survival, trying to get through each day with continued pandemic-related uncertainties. Still unknown are the longitudinal impacts of morally injurious experiences, as well as the relative impacts of one morally injurious experience as compared to prolonged and repetitive experiences. Future research in these areas is needed to further understand moral injury, delineate its psychosocial impacts, and inform appropriate interventions.

Findings of this research extend prior literature investigating moral injury among military personnel and healthcare professionals, and offer important insights for psychoeducation, screening, prevention, and intervention initiatives for frontline long-term care workers, as outlined in further detail below. Furthermore, our findings support the previously discussed literature suggesting that moral injury may be seen as a unique and separate, though related and overlapping experience to PTSD, which has critical implications for determining best treatment approaches. Specifically, recent studies have been calling for unique treatment methods, rather than the historical approach of using PTSD treatments given the major differences in symptomology. Our findings as well as those emergent from other studies also highlight the transdiagnostic features of moral injury, with associations to a range of psychiatric disorders, bolstering support for transdiagnostic treatment approaches. Farnsworth and colleagues (41) discussed the importance of using techniques such as Acceptance and Commitment Therapy (ACT) for treating moral injury, rather than the classic fear reduction techniques commonly used for treating PTSD. Cognitive therapy interventions for moral injury targeting rumination following exposure to morally injurious events, such as Ehlers and Clark's (16) cognitive model of PTSD, holds important possibility in addressing moral injury and associated symptoms (17).

Providing education on moral injury and associated symptoms, as well as opportunities for screening, could help to increase the understanding of this term among long-term care workers. Increased awareness may allow workers to feel less alone in their experiences and symptom and encourage access of available resources and supports. Furthermore, integrating group-based strategies such as Schwarts Rounds could provide a unique opportunity for all staff members in a long-term care facility to engage in meaningful discussions regarding the emotional challenges of their work and addressing moral injury in an organized and reflective space (42, 43). Findings from the current research confirm the importance of peer-led or peer-supported interventions, with participants stressing the importance of workplace team connection and discussions with those who understand frontline experiences in long-term care. This desire to be understood in an intervention is supported by previous work that has suggested individuals who experience moral injury may be particularly reluctant to access services due to concerns about possible legal consequences for disclosing their experiences (35). Additionally, other researchers have stressed the importance of creating treatment strategies that are organization based, due to the nature of morally injurious experiences (1). Creating a safe space where workers know they will not face legal ramifications for sharing their experiences may be particularly important among health care workers.

Findings also highlight the need for accessible, low-cost treatment options targeting moral injury in long-term care, which could take the form of virtual or e-health/m-health programming. One salient finding in terms of treatment access and barriers was that even with an abundance of webinars related to mental health, participants did not feel as though they had the capacity to engage. Completing ongoing evaluation of mental health programming for long-term care staff can help to ensure that available mental health resources are being appropriately targeted to meet needs. Based on the grounded theory model emergent from this work, in addition to clinical diagnostic findings, we have developed a preliminary conceptualization of an integrative treatment for moral injury in long-term care staff, to be further examined in future research. This six-session intervention consists of psychoeducation; institutional betrayal theory; cognitive therapy identifying and restructuring stuck points surrounding morally injurious events; connecting and reconnecting with valued purpose for long-term care work; taking care of the self in stressful times (mindfulness, emotion regulation); and cultivating continued support and valued action. Though beyond the scope of the current study, future research will seek to refine and validate this model with a broader sample of long-term care staff and management.

An important concept that has gained recent traction is institutional betrayal. This term was coined by researchers at the University of Oregon to describe the experience of someone being betrayed by an institution that they may have been a part of (44). Several participants in this study noted feelings of disappointment, betrayal, gaslighting, or neglect due to actions or inactions carried out by the management from their institution. This is an important concept when discussing moral injury within our population as institutional betrayal may compound or exacerbate the experience of moral injury, make workers feel unsafe in their workplace, and may isolate workers from seeking help from other colleagues. Given that many workers noted ethical difficulties with the adhering to the public health restrictions enforced by their institution, it is likely that institutional betrayal could be experienced by many in this line of work. Future work is warranted to explore institutional betrayal among long-term care workers.

This study is not without limitations, including sample homogeneity in terms of restricted range in cultural background. According to a study conducted in 2015, researchers found that over half of health care aids working in Canadian long-term care facilities were not born in Canada, and over 90% of the sample identified as female (45). According to 2016 Census Data from Statistics Canada, 31 and 34% of all nurse aides, orderlies and patient service associates (including health care and long-term care aides) identified as immigrant women and as belonging to a visible minority group, respectively (46). However, the majority of our sample reported European origins, limiting our understanding of the impact of moral injury in more culturally diverse and racialized groups. This is a critical issue given that the pandemic has highlighted health inequities that have disproportionate impacts on low-income, racialized and 2SLGBTQIA+ communities in Canada (47). Future research should seek to understand the compounding impact of marginalization and intersecting identities and experiences of moral injury within long-term care settings. Additionally, our sampling technique required participants to reach out to us first so we were unable to obtain response rates, and our sample may have some selection bias whereby we captured more participants who felt strongly connected to their line of work, in addition to participants who maintained their employment in long-term care during the pandemic (at the time of their interview and clinical assessment) as opposed to leaving or considering employment elsewhere. Though our study did not aim to be generalizable to all long-term care employees, it is important to note that our sample had elevated rates of self-reported current mental health problems (72%) and current use of mental health services (75%) which is elevated in comparison to the general population and likely does not extend to the broader population of Canadian long-term care employees.

However, our study also has several strengths. This study contributes to the growing body of literature investigating the psychological impacts of COVID-19. Particularly, our findings provide a wealth of knowledge on the experience and impact of moral injury on long-term care workers during this pandemic. Our future work aims to unpack how such experiences may get “under the skin” and translate into physiological effects on heart rate and sleep, as measured by fitness monitors worn by the frontline workers in this study. With long-term care facilities facing national scrutiny, it is important to amplify the voices and needs of the workers and ensure we are advocating for their needs to help them provide the best care they can. The pandemic has shed a light on the disastrous conditions within the long-term care sector and has provided an opportunity for changes to be made. We urge government, management, and people in authority in long-term care facilities to attend to the needs of their staff to provide a positive work experience so we can have sustainable, safe, and successful long-term care facilities in the future.

## Data Availability Statement

The raw data supporting the conclusions of this article will be made available by the authors, without undue reservation.

## Ethics Statement

The studies involving human participants were reviewed and approved by University of Manitoba Research Ethics Board. The patients/participants provided their written informed consent to participate in this study.

## Author Contributions

All authors listed have made a substantial, direct, and intellectual contribution to the work and approved it for publication.

## Funding

We acknowledge the funding received from the Government of Canada Department of National Defense (Innovation for Defense Excellence and Security -IDEaS: Moral Trauma on the Frontline: See, Prevent and Treat; KR, PI) and the Social Sciences and Humanities Research Council (Partnership Engage Grant; KR, PI). We acknowledge funds received through the University of Manitoba toward open access publication fees.

## Conflict of Interest

The authors declare that the research was conducted in the absence of any commercial or financial relationships that could be construed as a potential conflict of interest.

## Publisher's Note

All claims expressed in this article are solely those of the authors and do not necessarily represent those of their affiliated organizations, or those of the publisher, the editors and the reviewers. Any product that may be evaluated in this article, or claim that may be made by its manufacturer, is not guaranteed or endorsed by the publisher.
